# Subgingival microbiome of rheumatoid arthritis patients in relation to their disease status and periodontal health

**DOI:** 10.1371/journal.pone.0202278

**Published:** 2018-09-19

**Authors:** Kathrin Beyer, Egija Zaura, Bernd W. Brandt, Mark J. Buijs, Johan G. Brun, Wim Crielaard, Anne Isine Bolstad

**Affiliations:** 1 Department of Clinical Dentistry, Faculty of Medicine, University of Bergen, Bergen, Norway; 2 Department of Preventive Dentistry, Academic Centre for Dentistry Amsterdam, University of Amsterdam and VU University Amsterdam, Amsterdam, The Netherlands; 3 Department of Rheumatology, Haukeland University Hospital, Bergen, Norway; 4 Department of Clinical Science, University of Bergen, Bergen, Norway; VU University Medical Center, NETHERLANDS

## Abstract

**Objective:**

Rheumatoid arthritis (RA) and periodontitis are chronic inflammatory diseases that share common risk factors. However, the bidirectional relationship between RA and periodontal disease is not fully understood.

This study was undertaken to describe the bacterial component of the subgingival microbiome in RA patients and to relate this to RA disease activity and periodontal status.

**Methods:**

Patients with chronic established RA (N = 78) were periodontally examined and their subgingival plaque samples were collected; their clinical and laboratory data on RA status and medication were obtained from medical records. Bacterial DNA was quantified by universal 16S rDNA qPCR, and *Porphyromonas gingivalis* by species-specific qPCR. For microbiome assessment, 16S rDNA amplicon sequencing was performed.

**Results:**

Active RA was diagnosed in 58% of the patients and periodontitis in 82% (mild: 9%, moderate: 55%, severe: 18%). *P*. *gingivalis* was present in 14% of the samples. Different levels of gingival bleeding, periodontal probing depth, RA disease status, prednisolone use and smoking were associated with significantly different microbiome compositions. Two subgingival microbial community types were discerned.

**Conclusion:**

In RA patients with active disease, anti-inflammatory medication as part of RA therapy was associated with better oral health status and a healthier subgingival microbiome compared to that of RA patients in remission, especially those in remission who were current smokers. RA patients in remission with current smoking status may particularly benefit from a systematic periodontal treatment program. The potential role of microbial community types in patient stratification and personalized therapy should be assessed in longitudinal studies.

## Introduction

Rheumatoid arthritis (RA) and periodontitis are chronic inflammatory diseases that share complex multifactorial pathologic processes including environmental, inflammatory and genetic pathways [1, 2]. Evidence obtained from systematic reviews [3, 4] and meta-analyses [5] supports an association between both diseases. Dysregulation of the host inflammatory response is proposed as crucial underlying mechanism in both diseases [6].

Host-microbe interaction is essential for recognition and development of the immune system [7]. Imbalanced composition of the microbial community, called dysbiosis, has been related to the severity of both RA and periodontitis [1, 8]. Periodontopathogenic bacteria have been suggested to be involved in the loss of immune tolerance and development of RA [2, 9–11]. *Porphyromonas gingivalis*, one of the major periodontal pathogens, was found to deregulate local immune responses and to promote dysbiosis [12]. Anti-citrullinated protein antibodies (ACPAs) are highly specific for the diagnosis of RA [13]. The *P*. *gingivalis-*specific enzyme peptidyl-arginine-deiminase (PPAD) is capable of citrullinating human proteins. It has been speculated that *P*. *gingivalis* could contribute to generation of ACPAs in RA patients [14].

Detailed knowledge on the composition of the subgingival microbiome will increase our understanding of the biological mechanisms behind the association between RA and periodontitis and bring us one step further to personalized therapies. Therefore, the aim of this study was to describe the bacterial component of the subgingival microbiome in RA patients with different degrees of periodontal disease and to relate this to RA disease activity and periodontal status.

## Materials and methods

### Study design

The data for this cross-sectional study were collected between May 2013 and March 2016 at the Department of Rheumatology, Haukeland University Hospital, Bergen and Department of Clinical Dentistry, University of Bergen, Norway. The study protocol and written informed consent from all participants according to the Helsinki Declaration of 1975, version 2008 [15], were approved by the Institutional Medical Research Ethics Committee (2012/2212), University of Bergen, Norway.

### Study population

In this study, RA outpatients with chronic established RA were invited to participate. RA disease was classified using the 2010 classification criteria of American College of Rheumatology/European League Against Rheumatism (ACR/EULAR) [16]. Inclusion criteria were chronic established RA, Caucasian ethnicity and ≥ 35 years of age. The criteria for exclusion were diabetes, malignancy, pregnancy, breastfeeding and antibiotic use within 3 months prior to the study. Demographic and behavioral characteristics were collected using questionnaires. Past medical history, clinical and laboratory data on RA status and medication were obtained from medical records.

Recorded patient-related data included disease duration of RA, modified health assessment questionnaire (MHAQ) [17], RA disease activity score (DAS28), joint damage and patient global health assessment scored on a visual analogue scale (VAS). Routine laboratory analyses included erythrocyte sedimentation rate (ESR), C-reactive protein (CRP), rheumatoid factor (RF) and ACPAs. Based on the laboratory reference level, all values >25 IU/mL for RF and ≥ 3 U/mL for ACPAs were classified as seropositive for autoantibodies. Seropositivity has been defined as being tested positive for RF and/or ACPAs. RA disease activity score (DAS28) was calculated using tender 28 joint score, swollen 28 joint score, erythrocyte sedimentation rate (ESR) and VAS [18]. Active RA was defined as DAS28 ≥2.6 and RA disease in remission as DAS28 <2.6 [19]. Radiographic joints damage of hands and feet (destructive arthritis), as measured by the Van der Heijde modification of the Sharp score, were recorded as present or absent [20, 21]. Disease modifying anti-rheumatic drugs (DMARDs) were grouped as follows: conventional DMARDs (methotrexate, leflunomide, hydroxychloroquine, sulfasalazine) and biological DMARDs (tumor necrosis factor (TNF)-inhibitors, B-cell inhibitors, interleukin-6 (IL-6) inhibitors) and a combination of conventional and biological DMARDs.

The assessment of periodontal status was adapted from the Centers for Disease Control (CDC)-American Academy of Periodontology (AAP) clinical case definitions [22] with some modifications. Subjects were classified into four sub-groups: 1) gingivitis: probing depth (PD) ≤3 mm and bleeding on probing (BoP); 2) mild periodontitis: ≥ 2 interproximal sites with CAL ≥3 mm, and ≥ 2 interproximal sites with PD ≥4 mm (not on the same tooth) or one site with PD ≥5 mm, and BoP; 3) moderate periodontitis: ≥ 2 interproximal sites with CAL ≥4 mm (not on the same tooth), or ≥ 2 interproximal sites with PD ≥5 mm (not on the same tooth), and BoP; or 4) severe periodontitis: ≥ 2 interproximal sites with CAL ≥ 6 mm (not on the same tooth) and ≥ 1 interproximal site with PD ≥ 5 mm and BoP.

The level of oral hygiene practices and dental-visit habits was assessed by an oral hygiene questionnaire (OHQ).

Oral impacts on eight physical, social and psychological aspects of daily living (OIDP) were assed using a validated OIDP questionnaire [23]. The OIDP was measured as a count score and dichotomized into no impacts (score = 0) and one or more impacts (score = 1).

Current smokers were defined as subjects who smoked or stopped smoking less than 12 months prior to being enrolled into the study. Former smokers were subjects who quit smoking more than 12 months ago.

### Clinical oral examination

Clinical oral examination and periodontal data collection were performed under standardized conditions by a single calibrated dentist (KB). Detailed description is provided in Supplementary materials and methods (S1 Supplementary materials and methods).

Prior to periodontal examination, RA patients were instructed not to perform any oral hygiene measures at the morning before the appointment. A comprehensive periodontal examination including registration of PD, CAL, BoP and accumulation of dental plaque (PI) at six sites per tooth was assessed using a manual periodontal probe (PCP-26, Hu-Friedy^®^, Chicago, IL, USA). The BoP, registered as present or absent, was assessed as a modification described previously [24]. Dental plaque was stained with fluorescent disclosing solution (Plaque Test, Ivoclar Vivadent AG, Liechtenstein) and visualized using ultraviolet light (Satelec Mini LED, Kaltenbach & Voigt GmbH, Germany) according to manufacturer’s recommendations. The PI was calculated as percentage of stained tooth surfaces with biofilm [25].

Salivary flow rate (SFR) of unstimulated (US) and stimulated (SS) whole saliva was measured during the morning hours before dental examination as described by Navazesh [26]. The patients were asked not to eat, drink and smoke for at least two hours prior to sampling. Saliva samples were collected by continuously spitting into a sterile, pre-weighed collection tube (50 ml, Sarstedt AG & Co, Germany). The US saliva was collected for 15 min, while the SS was collected for 5 min by chewing a paraffin pellet (Ivoclar Vivadent AG, Liechtenstein). The SFR rate was measured as mg/min.

### Subgingival plaque sampling

Microbial sampling was undertaken at the three deepest interproximal sites of non-adjacent teeth in every patient. Sampling in three out of four quadrants was conducted wherever possible. In case of multiple sites with equally deep PD, sampling sites were selected in order to assure proper sampling requirements after the following criteria: accessibility for supragingival plaque removal, prevention of saliva contamination and assurance of proper curette angulation. After isolation with cotton rolls, the tooth was air dried and supragingival biofilm was removed with sterile cotton pellets using forceps. Then a sterile curette (LM-ErgoMix, LMDental, Finland) was gently inserted into the pocket, moved down to the base of the probable pocket without tooth contact and then angled to get in close contact to the tooth root surface to scrape off the biofilm. A sterile dental explorer (Hu-Friedy Mfg. Co., IL, USA) was used to remove the subgingival plaque from the curette. The plaque was stored in a DNAse/RNase-free cryovial (Biosphere^®^ SC Micro Tube 2.0ml, Sarstedt AS & Co., Germany) containing 1 ml of RNAlater (Ambion Inc, TX, US). The samples from the three sites were pooled. For each pocket, a new sterile curette was used. The samples were stored at -80°C until processing.

### Sample processing, 16S rDNA amplicon sequencing and *P*. *gingivalis* quantification

Detailed sample processing and sequencing is described in Supplementary material and methods (S1 Supplementary material and methods). In brief, DNA was extracted using the Mag Mini DNA Isolation Kit with 0.1-mm Zirconia beads in a Mini BeadBeater. Bacterial DNA was determined by 16S rDNA quantitative polymerase chain reaction (qPCR) [27]. The V4 hypervariable region of the 16S rRNA gene was amplified as described previously [28] with the adaptation that we performed 33 amplification cycles. The sequencing was conducted on the MiSeq platform (Illumina, San Diego, CA, USA). *P*. *gingivalis* was quantified by qPCR as described previously [29].

### Sequencing data processing

Detailed data processing is described in Supplementary material and methods (S1 Supplementary material and methods). In brief, the sequencing reads were merged, quality filtered and clustered into operational taxonomic units (OTUs) using USEARCH [30]. QIIME [31] and the RDP classifier [32] with the SILVA database [33] were used to assign taxonomy to OTUshttps://link.springer.com/article/10.1007%2Fs00248-016-0775-z-CR46. The Human Oral Microbiome Database (HOMD) [34] was used to further classify the OTUs. The OTU table was randomly subsampled at an equal depth per sample using QIIME.

### Statistical analyses

The distribution of the variables was tested using the Shapiro-Wilk test for normality. The Wilcoxon rank-sum test was applied for not normally distributed continuous variables. The Student’s t-test (for continuous variables) and the Pearson chi-square test (for categorical variables) were used to assess differences between the groups. These analyses were performed in STATA version 14.0 for Microsoft Windows (StataCorp LP, Texas, USA).

Nonmetric multidimensional scaling (nMDS) plots based on Bray-Curtis distance were used to visualize similarity between groups of samples. One-way permutational multivariate analysis of variance (PERMANOVA) with the Bray-Curtis distance measure was used to assess differences in microbiome profiles among different groups of categorical variables and among the groups obtained from continuous variables categorized into tertiles (lowest, middle and highest tertile). In case of multiple comparisons, the p-value was corrected using Bonferroni correction. Canonical correspondence analysis (CCA) was used to visualize the relation of the microbiome composition with different continuous variables [35]. To assess microbiome diversity, the Shannon Diversity index was used. All above analyses were performed in PAST [36].

Linear Discriminant Analysis Effect Size (LEfSe) was used to determine which OTUs contributed to the observed significant differences among different groups of samples [37]. Only OTUs with at least 100 reads were included in the analyses.

Assessment of significant patterns of microbial co-occurrence or mutual exclusion at the genus or higher taxonomic level was performed using CoNet [38] and visualized in Cytoscape. A dataset of relative abundances of reads at the genus level including the 52 most abundant (average abundance 0.05% or above) genera or higher taxa, and the remaining taxa collectively termed “Others”, was used as described by Faust et al. [38].

## Results

The study population (N = 78, 73% females), aged 57 ± 11.5 years, consisted of 29 never smokers, 14 current smokers, and 35 former smokers. A detailed description of the study subjects (demographic, behavioral and clinical characteristics) is presented in Beyer et al. [39] and in S1 and S2 Tables. Periodontitis was diagnosed in 64 individuals (mild periodontitis: N = 7, moderate periodontitis: N = 43, severe periodontitis: N = 14). The remaining 14 individuals were diagnosed with gingivitis.

Active RA disease was diagnosed in 45 patients.

Analysis of OHQ revealed a high level of daily oral hygiene performance: all patients performed daily tooth brushing, of them 90% brushed their teeth twice a day or more. Daily interdental cleaning was performed by 95% of the patients using toothpicks (64%), dental floss (58%) and/or interdental brushes (31%). Furthermore, regular dental visits were reported by 91% of the patients, of them 90% had recall intervals between 6 and 12 months, 4% had intervals between two to four months.

SFR of unstimulated and stimulated whole saliva showed a mean and standard deviation of 0.34 ± 0.28 and 1.88 ± 0.88 respectively (S2 Table). Unstimulated SFR was found to be low (< 0.10 mg/g) in ten patients, none of the included RA patients has been diagnosed with Sjögren’s syndrome.

### Microbial sampling

In our cohort, periodontitis sites were evenly distributed between never, former and current smokers, although smokers had higher mean PD (3.1 ± 0.5 mm) compared to never smokers (PD: 2.6 ± 0.2 mm, *p*<0.01) and former smokers (PD: 2.8 ± 0.5 mm, *p* = 0.025). The highest mean PD and CAL, reported as mean and 95% confidence interval (95% CI), were located in mandibular molars (never smokers: PD 3.0 (3.0–3.1) mm /CAL 3.3 (3.2–3.4) mm; former smokers: PD 3.3 (3.2–3.5) mm /CAL 3.7 (3.4–4.1) mm; current smokers; PD 3.6 (3.4–3.9) mm /CAL 4.0 (3.7–4.3) mm) followed by maxillary molars (never smoker: PD 2.8 (2.7–2.8) mm /CAL 3.1 (3.0–3.2) mm; former smokers: PD 3.1 (2.9–3.2) mm/CAL 3.6 (3.2–3.9) mm; current smokers; PD 3.3 (3.1–3.5) mm/CAL 3.8 (3.5–4.1) mm). Microbial sampling in anterior sites was undertaken equally in never, former and current smokers, accounting for 17% of the sampled sites. The mean PD (4.4 ± 1.0 mm) of the sampling sites was significantly higher compared to mean PD at patient level (2.8 ± 0.4 mm, *p*<0.01).

### Overall sequencing results and microbiome profile analyses

Out of 4 million sequences, 54% of the raw read pairs showed exact overlaps (i.e. without any mismatch) and 90% of the raw read pairs passed the merging and the stringent quality-filtering step. After quality control, the average sequencing depth was 19784 (SD 4004) reads/sample (median 20107, range: 3818–28361). The dataset, subsampled to 5000 reads/sample, contained 552 OTUs with 97 ± 21 OTUs (min 40, max 145) per sample (S3 Table). The OTUs were classified into 13 phyla or candidate divisions, with Bacteroidetes (125 OTUs, 26% of all reads), Fusobacteria (37 OTUs, 22% of reads), Actinobacteria (95 OTUs, 18% of reads), Firmicutes (169 OTUs, 17.6% of reads), Proteobacteria (66 OTUs, 8.5% of reads) and Spirochaetae (26 OTUs, 4.9% of reads) dominating the dataset, while 0.02% of the reads could not be classified beyond domain bacteria. Further taxonomic classification of the OTUs resulted in 124 genera or higher taxa. The most predominant genera were *Fusobacterium* (16% of all reads), *Prevotella* (14.5%), *Corynebacterium* (8.2%), *Actinomyces* (6%), *Leptotrichia* (5.5%), *Selenomonas* (5.3%), *Veillonella* (5%) and *Treponema* (4.9%).

**Fig 1:**
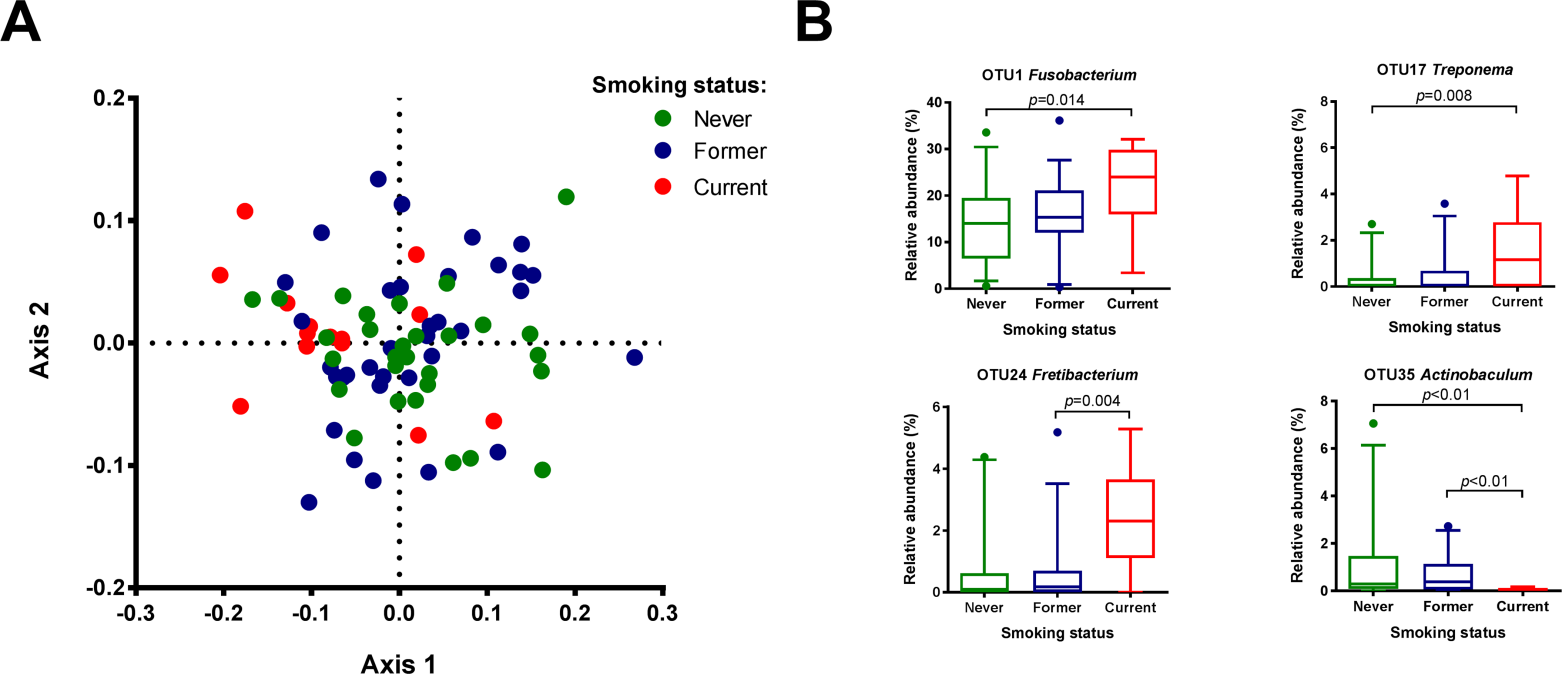
Association between smoking status and subgingival microbiome of rheumatoid arthritis (RA) patients. (A) Nonmetric multidimensional scaling (nMDS) plot based on two-dimensional Bray-Curtis similarity index (stress 0.1991, PERMANOVA *p* = 0.0017, F = 2.3). Pairwise comparisons: never smokers vs current smokers: *p* = 0.0033, former smokers vs current smokers: *p* = 0.0048 (*p* values after Bonferroni correction for multiple comparisons). Never smokers (N = 29) = green dots; Former smokers (N = 35) = blue dots; Current smokers (N = 14) = red dots. (B) Boxplots of the most abundant 37 OTUs (S4A Table) that were significantly associated with smoking status by linear discriminant analysis effect size (LEfSe) analysis. P values are based on Wilcoxon rank-sum test after Bonferroni correction for multiple comparison. The boxplots show medians, the error bars indicate 5–95% confidence interval. The connectors show statistically significant differences (*p*<0.05).

Of the demographic and behavioral variables tested (age, gender, BMI, smoking status), only the smoking status showed a significant association with the microbiome profile of subgingival plaque (*p =* 0.0017, F = 2.3) (Fig 1A). Microbiomes of current smokers differed significantly from never-smokers (*p* = 0.0033, after Bonferroni correction) and former smokers (*p* = 0.0048, after Bonferroni correction). The biomarker discovery tool LEfSe identified 37 OTUs that discriminated between these groups (S4A Table). Never-smokers and former smokers contained higher proportions of aerobic and facultative anaerobic taxa such as *Neisseria*, *Haemophilus*, *Corynebacterium* and *Capnocytophaga* in their subgingival plaque, while the microbiome of current smokers had a higher proportion of anaerobes such as *Fusobacterium*, *Synergistaceae*, *Fretibacterium*, *Paludibacter* and *Treponema*, than the other two groups of samples (Fig 1B, S4A Table). There were no differences in microbiome diversity estimates by any of the clinical variables above (data not shown).

### Subgingival microbiome by oral health parameters

Next, we assessed the relation between periodontal status and subgingival microbiome composition. No significant differences in microbiome profiles were found in relation to the periodontal diagnosis of gingivitis, or mild, moderate or severe periodontitis (PERMANOVA, *p* = 0.716). However, a significant relation was found between the microbiome profiles and mean PD (*p* = 0.038, F = 1.6), BoP (*p* = 0.008, F = 1.9) and PI (*p* = 0.029, F = 1.7).

**Fig 2:**
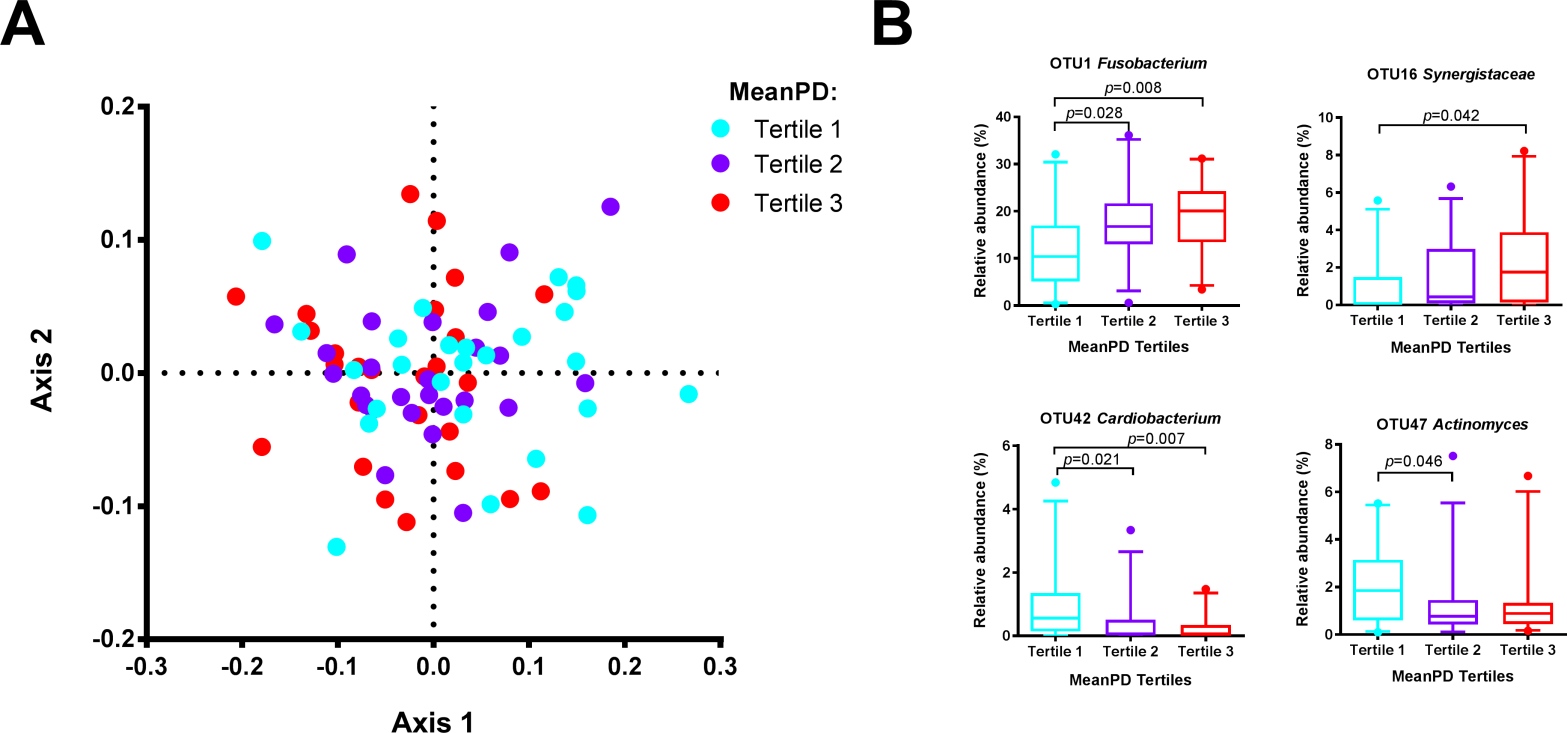
Association between mean probing depth (PD) and subgingival microbiome of rheumatoid arthritis (RA) patients. (A) nMDS plot based on two-dimensional Bray-Curtis similarity index (stress 0.1982, PERMANOVA: *p* = 0.038, F = 1.6). Pairwise comparisons: the samples with the deepest probing depths (PD tertile 3) vs the samples with the shallowest probing depths (PD tertile 1): *p* = 0.039. Samples in the lowest tertile of PD (2.2–2.5 mm) = aqua dots; samples in the middle tertile of PD (2.6–2.8 mm) = purple dots; highest tertile of PD (2.8–4.4 mm) = red dots. (B) Boxplots of the most abundant and significant 20 OTUs (S4B Table) that significantly associated with mean PD by linear discriminant analysis effect size (LEfSe) analysis. P values are based on Wilcoxon rank-sum test. The boxplots show medians, the error bars indicate 5–95% confidence interval. The connectors show statistically significant differences (*p*<0.05).

The microbiomes of the lowest tertile of PD differed significantly from the microbiomes of the samples with the highest PD (*p* = 0.039) (Fig 2A). LEfSe identified 26 OTUs that discriminated between the samples in these two tertiles (S4B Table). The microbiome in shallow pockets had a significantly higher proportion of facultative anaerobes and capnophilic taxa such as *Actinomyces gerencseriae* (OTU47), *Cardiobacterium hominis* (OTU42), *Corynebacterium durum* (OTU44), *Aggregatibacterium segnis*/Oral Taxon (OT) 458/512 (OTU253), *Capnocytophaga sputigena* (OTU46) and *Capnocytophaga haemolytica* (OTU116), while deep pockets were associated with anaerobic taxa such as *Fusobacterium* (OTU1), *Fretibacterium* OT 360 (OTU16), *Fretibacterium fastidiosum* (OTU24), *Tannerella forsythia* (OTU27), *Treponema denticola* (OTU13) and *Selenomonas* (OTU31) (Fig 2B, S4B Table).

Similarly to the PD, microbiomes of the samples grouped into the lowest tertile of the PI differed significantly from the microbiomes of the samples with the highest PI (*p* = 0.023). Among the 27 OTUs that discriminated the two groups of samples, streptococci (OTU8, OTU232), *Rothia dentocariosa* (OTU15), *Actinomyces naeslundii* /*oris* / OT169/171/175 (OTU22), *Actinomyces gerencseriae* (OTU47), *Corynebacterium durum* (OTU44), *Kingella oralis* (OTU36) and *Cardiobacterium hominis* (OTU42) were associated with low visible (supragingival) plaque amount (S4C Table, S1 Fig).

The samples from the highest tertile of the bleeding scores (BoP) differed significantly from the samples with the lowest (*p* = 0.029) and from the samples with the moderate (*p* = 0.031) BoP. In total, 21 OTUs discriminated significantly among the tertiles (S4D Table). Samples with the highest bleeding scores had the highest proportion of strict anaerobes such as *Alloprevotella tannarae* (OTU20), *Fretibacterium* (OTU16, OTU24) and *Treponema denticola* (OTU13) (S2 Fig).

There were no differences in microbiome diversity (Shannon Diversity index) by any of the periodontal variables above (data not shown).

### Subgingival microbiome by RA status and treatment

**Fig 3:**
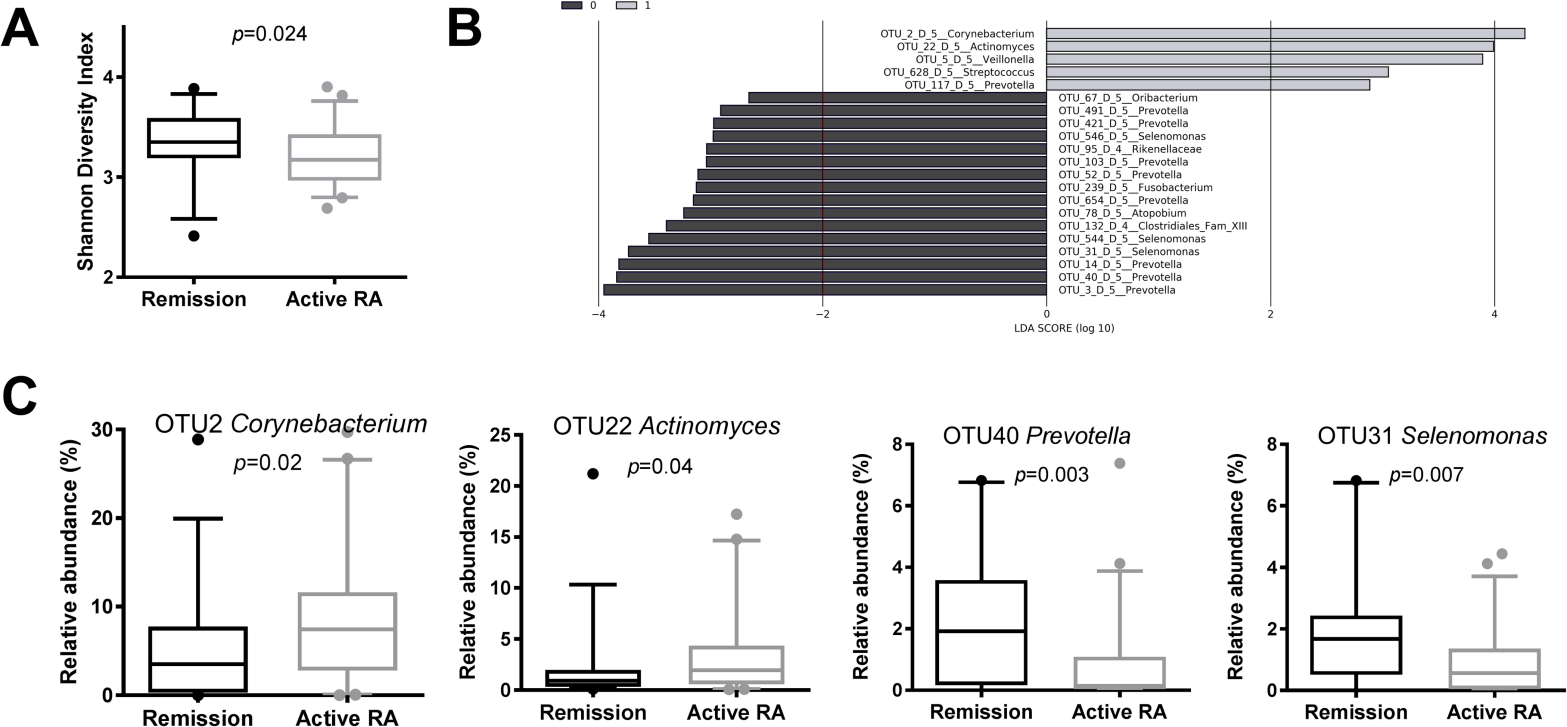
Association between RA disease activity status and subgingival microbiome. (A) Shannon Diversity index by RA activity status. DAS28 score ≥2.6 = individuals with active RA (N = 60), DAS28 score <2.6 = individuals in RA remission (N = 28). P value is based on Mann-Whitney test. (B) Differentially discriminatory OTUs (N = 21) by RA disease activity status, as identified by linear discriminant analysis effect size (LEfSe) analysis. Active RA = grey bars; RA remission = black bars. (C) Boxplots of the most abundant and significant 21 OTUs (S4E Table) that significantly associated with RA disease activity status by LEfSe analysis. *P* values are based on Wilcoxon rank-sum test. Active RA = grey boxplots; RA remission = black boxplots. The boxplots show medians, the error bars indicate 5–95% confidence interval. The connectors show statistically significant differences (*p*<0.05).

The activity of RA correlated negatively with the diversity of subgingival microbiome (*p* = 0.007, R = -0.3026, Spearman’s correlation). Although microbiomes from individuals with active disease (DAS28 score ≥2.6) had lower Shannon Diversity index *p* = 0.024, Mann-Whitney test) than the microbiomes from individuals in remission (DAS28 score <2.6) (Fig 3A), the difference in microbiome profiles between the two groups did not reach statistical significance (*p* = 0.07, PERMANOVA). However, when the contribution of individual OTUs was assessed, 19 OTUs discriminated between samples from individuals with active RA and RA in remission (Fig 3B and 3C, S4E Table). For instance, microbiomes from individuals with active RA had higher proportion of *Corynebacterium matruchotii* (OTU2), *Actinomyces* (OTU22), *Veillonella* (OTU5) and *Streptococcus* (OTU628) than individuals with RA disease in remission.

Similarly to active RA state, the subjects who were diagnosed with destructive arthritis had subgingival microbiome with lower diversity than subjects without joint damage (*p* = 0.021, Mann-Whitney test). However, no differences in microbial composition could be found (*p* = 0.2, PERMANOVA).

**Fig 4:**
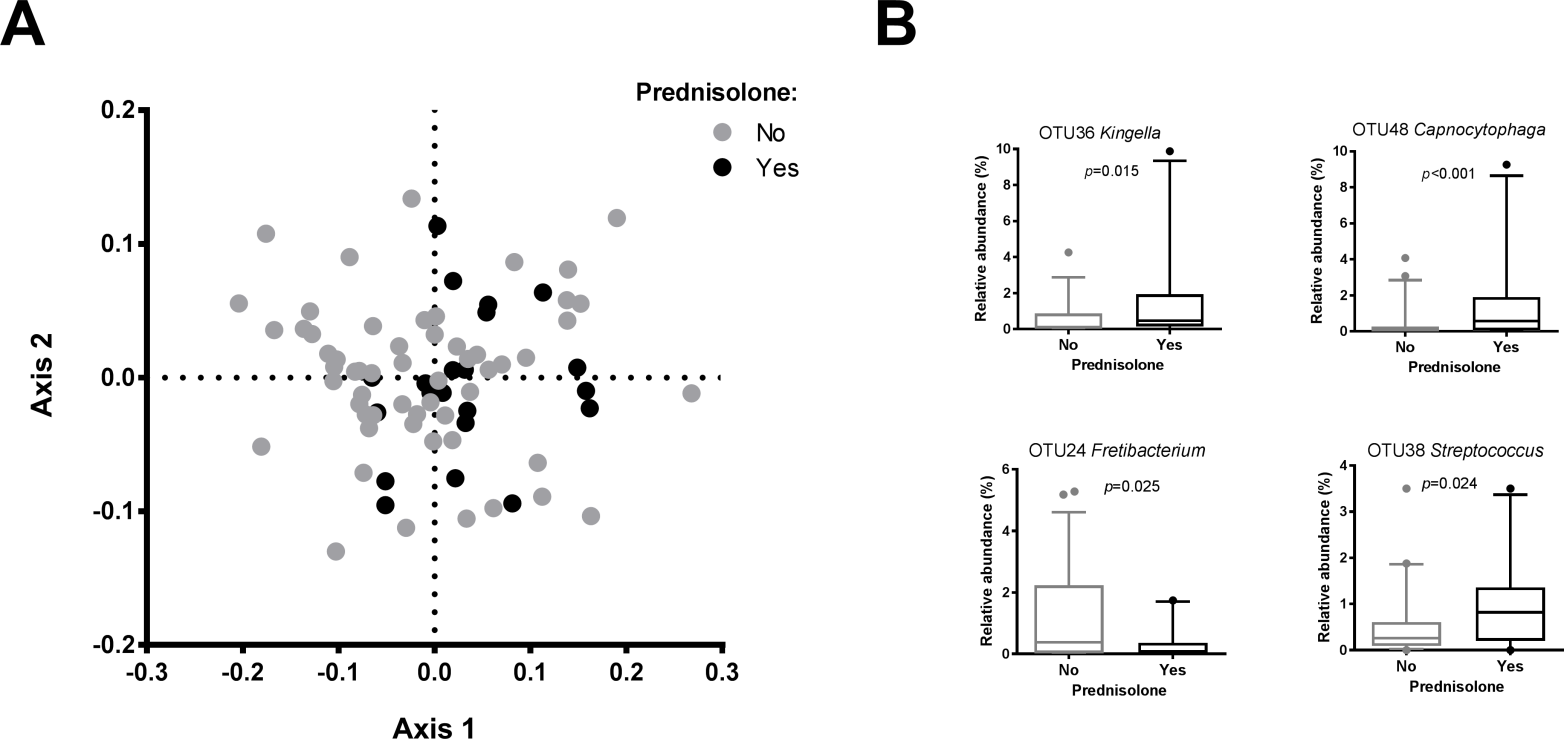
Association between prednisolone use and subgingival microbiome in RA patients. (A) nMDS plot based on two-dimensional Bray-Curtis similarity index (stress 0.1981, PERMANOVA: *p* = 0.045, F = 1.76). Prednisolone (N = 21) = black dots; no prednisolone (N = 57) = grey dots. (B) Boxplots of the most abundant and significant 19 OTUs (S4F Table) that significantly associated with use of prednisolone by LEfSe analysis. P values are based on Wilcoxon rank-sum test. The boxplots show medians, the error bars indicate 5–95% confidence interval. The connectors show statistically significant differences (*p*<0.05).

Next, we aimed at assessing the effects of anti-rheumatoid arthritis medication on the subgingival microbiome composition. Only four individuals did not receive any DMARDs, while the majority of the subjects used a combination of different types of medication of unspecified dosage (S1 Table). No difference in microbiome profiles among the DMARD groups (conventional, biological or both) was observed, while the microbiomes of the four individuals without DMARDs did differ from the group that used conventional DMARDs (*p* = 0.02, F = 2.02, PERMANOVA). Two individuals who did not receive any DMARDS did receive a broad-spectrum anti-inflammatory corticosteroid drug, prednisolone, while 19 individuals received prednisolone additionally to DMARDs. There was a significant difference between the subgingival microbiome profiles of individuals who received prednisolone and those who did not (*p* = 0.045, F = 1.76, PERMANOVA) (Fig 4A). At an individual OTU-level, 19 OTUs discriminated between these groups (S4F Table). The OTUs that were associated with prednisolone usage belonged mostly to facultative anaerobes and capnophilic taxa, while those without prednisolone use had higher proportions of strict anaerobes such as *Fretibacterium fastidiosum* (OTU24) and *Treponema lecithinolyticum* (OTU17) (Fig 4B and 4C). No relation between the microbiome diversity and the use of DMARDs or prednisolone was found (data not shown).

None of the other patients with RA associated variables (RF, ACPAs, and seropositivity for both, CRP, ESR, VAS, MHAQ) showed a significant relationship with microbiome composition or diversity.

### *P*. *gingivalis* in subgingival microbiome in relation to oral health and RA

To assess the relation of *P*. *gingivalis* with the study variables, we performed a *P*. *gingivalis*-specific qPCR and calculated the relative abundance of *P*. *gingivalis* over total bacterial DNA (16S rDNA). This analysis showed that only 11 (14%) samples were *P*. *gingivalis*-positive, with a median relative abundance of 2.7% (range 0.03–17%) in these 11 samples (N = 8 moderate and N = 3 severe periodontitis cases). The presence of *P*. *gingivalis* was associated with deeper PD and more severe BoP. Mean PD (*p* = 0.002, Mann-Whitney test), mean CAL (*p* = 0.002), sampled site-specific PD (*p* = 0.001) and BoP (*p* = 0.047) were higher in *P*. *gingivalis*-positive samples. Higher CRP was found in subjects with *P*. *gingivalis* in their biofilm than in those without (*p* = 0.009).

In the microbiome dataset, OTU9 was classified as *P*. *gingivalis* and was present in 21.8% of the patients. *P*. *gingivalis* quantification by both methods showed good correlation (*p*<0.001, R = 0.761, Spearman’s correlation (S3 Fig).

### Integration of oral health status and RA disease parameters with the microbiome profiles

**Fig 5:**
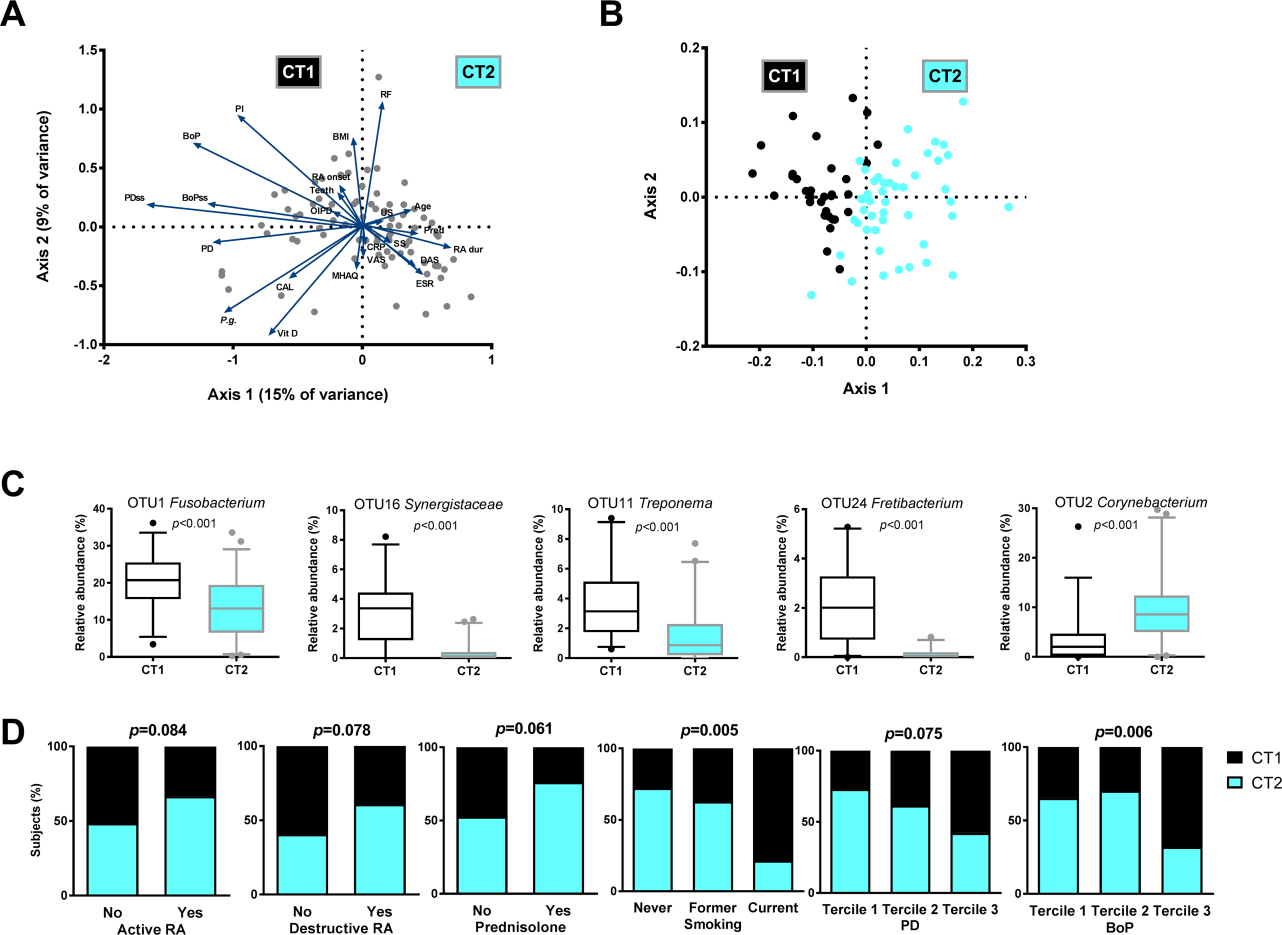
Integration of oral health status and rheumatoid arthritis (RA) disease parameters with the microbiome profiles. (A) Canonical correspondence analysis (CCA) ordination biplot, visualizing variation between subgingival microbiome samples and their association with 23 oral health-related and RA disease-related continuous variables (S1 and S2 Tables and Beyer et al. [39]). Only the first axis, explaining 15% variance, was statistically significant (*p* = 0.039). (B) nMDS plot by community type CT1 = black dots and CT2 = aqua dots, based on two-dimensional Bray-Curtis similarity index (stress 0.1994, PERMANOVA: *p* = 0.001, F = 13.75). The samples were dichotomized into community types according to their position in CCA (Fig 5A): Samples positioned to the left of the centre of the plot were grouped into microbial community type 1 = CT1, the samples to the right of the centre–into microbial community type 2 = CT2. (C) Boxplots of the most abundant and significant 113 OTUs (S4G Table) that discriminated between the two community types by LEfSe analysis. P values are based on Wilcoxon rank-sum test. The boxplots show medians, the error bars indicate 5–95% confidence interval. The connectors show statistically significant differences (*p*<0.05). (D) The strongest associations between microbial community type and oral health and RA variables. P values results from Pearson chi-square test.

Next, we aimed at a simultaneous assessment of relation among all continuous oral health and RA-related variables with the subgingival microbiome of our study population. For this, a Canonical Correspondence Analysis (CCA) was performed using 23 variables, of which 18 are listed in the S2 Table and the remaining five comprised of the relative abundance of *P*. *gingivalis*, the dose of prednisolone, the OIDP scores and the sampled site-specific PD and BoP values (Fig 5A). Only the first axis (x-axis in Fig 5A), explaining 15% variance, was statistically significant (*p* = 0.039). Based on these results, we dichotomized the samples according to their position on this axis: samples positioned to the left of the centre of the axis were grouped into microbial community type I (CT1), and the samples to the right of the centre–into microbial community type II (CT2). The difference between the microbiome profiles of these groups was highly significant (*p* = 0.0001, F = 13.75) (Fig 5B). At the OTU-level, 113 OTUs discriminated between the two community types (S4G Table). The CT1 was associated with higher proportion of taxa such as *Fusobacterium* (OTU1), *Fretibacterium* (OTU16, OTU24), *Prevotella* (OTU21, OTU3, OTU25), *Treponema* (OTU11, OTU13) and *Porphyromonas* (OTU6), while *Corynebacterium* (OTU2, OTU44), *Veillonella* (OTU5, OTU245), *Actinomyces* (OTU22, OTU47), *Leptotrichia* (OTU29, OTU39), *Streptococcus* (OTU8, OTU232) and *Neisseria* (OTU18) were associated with the CT2 (Fig 5C). There was no difference in microbial diversity between the two community types (*p* = 0.123).

Among the 23 variables, the CT1 samples correlated with decreased oral health status of the individuals (higher PD, BoP, PI), while the CT2 samples related to higher RA disease duration and disease activity, higher prednisolone dose and ESR (Fig 5A and 5D). Additionally, smoking (not included in CCA) was significantly associated with the CT1 (Fig 5D).

**Fig 6:**
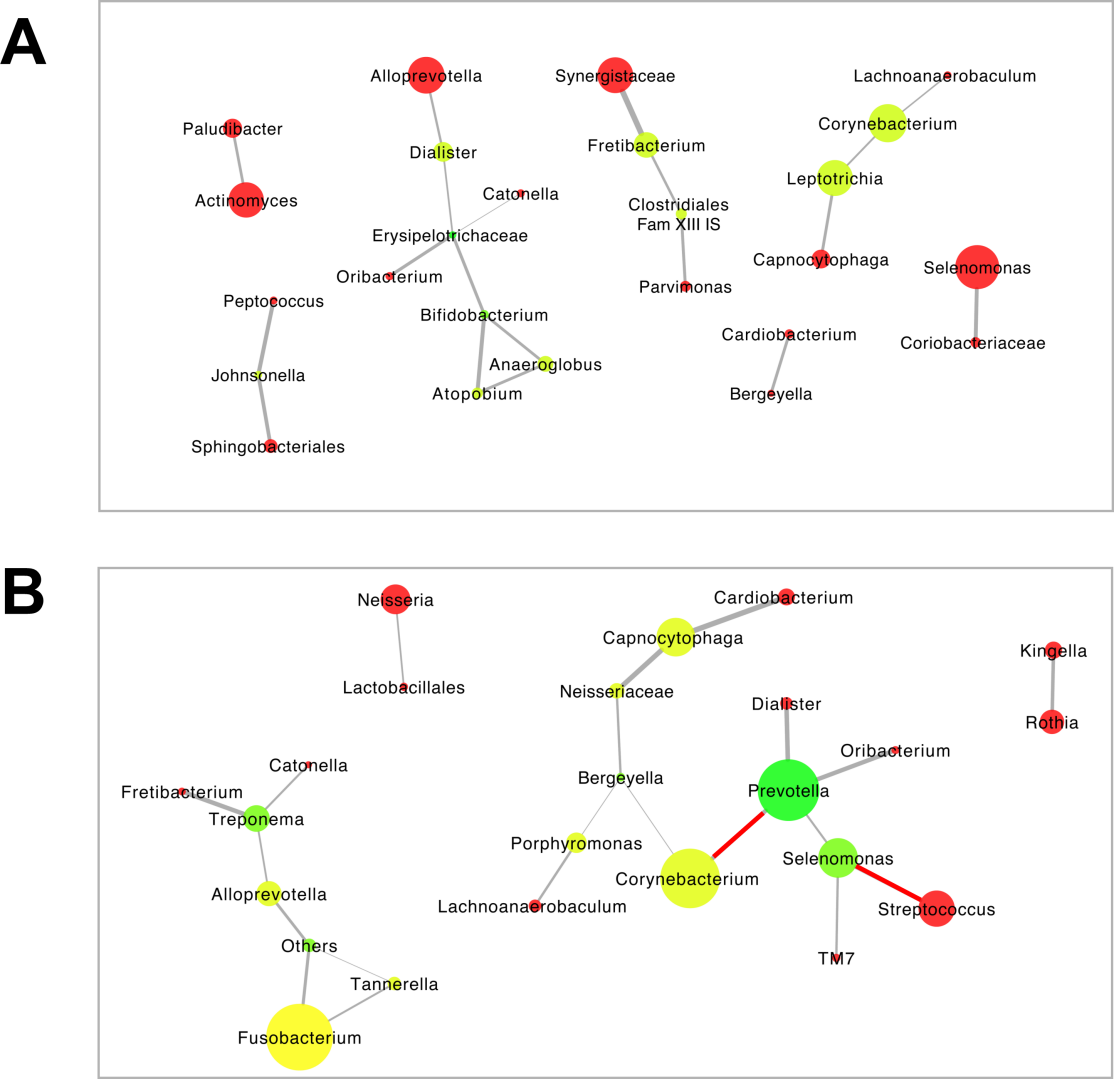
Bacterial co-occurrence or mutual exclusion networks by subgingival microbial community type in RA patients. (A) community type 1 = CT1. (B) community type 2 = CT2. (S1 and S2 Tables). Only edges that were significant by at least two of the methods (i.e. Pearson and Spearman correlations, Bray-Curtis similarity and Kullback-Leibler divergence) after correction for multiple comparisons were included in the network. The grey lines indicate positive correlations, and the negative correlations are indicated in red (*p* < 0.05). The size of the node is indicative of the relative abundance of the respective genus. The color of the node indicates the number of connections (neighbors) in the range of red to green; red indicates one connection, while green indicates ≥3 connections.

Thereafter, we assessed the structure of the mutual correlations among the most prevalent taxa within each of the community types (Fig 6). Both community types had a similar number of co-occurring taxa (25 and 24, respectively), while the CT1 had lower average number of neighbors (1.52) than the CT2 (1.75). All taxa in the CT1 network were co-occurring (Fig 6A), while in the CT2 two of the interactions were mutually exclusive: Genus *Streptococcus* was inversely associated with genus *Selenomonas*, and genus *Corynebacterium*—with genus *Prevotella* (Fig 6B).

## Discussion

This study investigated the bacterial component of the subgingival microbiome in RA patients with different periodontal conditions and related these findings to RA disease activity and periodontal health status of these individuals. The subgingival microbiome of subjects with active RA disease differed from those with the disease in remission. Besides disease activity, use of anti-inflammatory drug prednisolone, current smoking and periodontal status had significant impact on microbiome composition. Integration of microbiome profiles with oral health and RA parameters resulted in two compositionally distinct microbial community types.

Compared to RA patients in remission, patients with active disease and those who used prednisolone had lower bacterial diversity and a higher proportion of taxa which usually has been associated with supragingival plaque and periodontal health [40]. Since patients with active disease had significantly higher use of prednisolone than those in remission, the effect observed on oral ecology could be due to the use of this anti-inflammatory drug and not related to activity of the disease per se. However, in a study on treatment-naïve RA patients a positive correlation was found between disease activity and a single microorganism, namely *Lactobacillus salivariu*s [8], suggesting a role of this bacterium in the disease process of that particular group of patients. Some periodontopathogenic OTUs, such as *Tannerella* and *Treponema*, have been found in higher proportion in new-onset RA patients than in patients with chronic RA, and these differences was attributed to therapeutic immunomodulatory regimens over time in chronic RA patients [41].

To the best of our knowledge, there are no previous reports on alterations in oral subgingival microbiome as a result of oral administration of glucocorticoids. Our findings might be explained by the mechanism of action of prednisolone. It is a synthetic corticosteroid with predominant glucocorticoid and low mineralocorticoid activity, and is used as an anti-inflammatory agent [42]. Glucocorticoids inhibit the generation of glucocorticoid-sensitive cytokines and thereby prevent different aspects of inflammation, including the activation and recruitment of inflammatory cells (eosinophils, basophils, and lymphocytes) and the release of inflammatory mediators [43]. Thus, our observation on microbiologically healthier subgingival niche in prednisolone recipients might be explained by a cascade of processes leading to reduction of inflammation. The observed microbiological differences imply that patients exposed to prednisolone should have better periodontal health with less inflammation.

However, there was no significant association between the use of prednisolone or RA disease activity and the periodontal status (data not shown). This discrepancy might be due to low sensitivity of periodontal indices compared to changes in microbiological composition of subgingival plaque that are preceding the macroscopically detectable clinical signs.

In the present study, we have focused on subgingival microbiome of RA patients with different periodontal status. As expected, subgingival microbiome composition reflected PD, bleeding and plaque amount. Our finding is in accordance with a recent study using multiplexed-454 pyrosequencing, where the identified subgingival microbiome profiles were similar in patients with new-onset RA and chronic RA with comparable periodontal disease severity (41). However, in line with another recent study using PCR analysis [44], we did not find a significant relation between periodontal diagnosis and microbiome composition. The CDC-AAP periodontal status assessment has been developed to determine the prevalence of periodontitis in populations and has been described as conservative and underestimating periodontal disease [22]. Our findings suggest that this classification does not relate to site-specific differences in subgingival microbiome composition. Furthermore, the accuracy of present periodontal disease classification has been described as low [45].

Due to the proposed involvement of a specific periodontal pathogen, *P*. *gingivalis*, in RA disease, we targeted this microbial species by quantitative species-specific PCR.

The reason for the lower prevalence of *P*. *gingivalis* (14%) in this cross sectional study is not clear, but there are other studies showing similar results. For instance, our findings correspond with the results of de Smith et al., who found *P*. *gingivalis* in 16% of RA patients and 20% of non-RA patients [46]. Compared to our data, Ziebolz et al. found a higher prevalence of *P*. *gingivalis* (58%) in RA patients with comparable severity of periodontitis, although this study used a different classification system of periodontal disease [44]. Scher et al. found a higher prevalence of *P*. *gingivalis* in chronic RA patients (47%), especially in patients with advanced periodontal disease [41]. Interestingly, a more recent study using the CDC-AAP periodontitis case definitions and PCR techniques for microbial analysis did not find any statistical difference in prevalence of *P*. *gingivalis* in RA patients compared with healthy controls [47]. The discrepancy between mean PD at patient level and mean PD at microbial sampling sites supports the fact that periodontitis is known as a site-specific disease and shows unequivocally the need for thorough periodontal assessment to be able to evaluate extent and severity of periodontal inflammation and attachment loss.

The presence of *P*. *gingivalis* has been related to ACPAs [48]. However, we did not find any association between RA disease parameters and *P*. *gingivalis* except for CRP, which is used as part of DAS28 score but is also a marker of non-specific systemic inflammation. This is in line with a recent study on new-onset and chronic RA patients where *P*. *gingivalis* presence and abundance did not correlate with ACPA titers [41]. Other studies on RA found an increased expression of ACPAs in the presence of *P*. *gingivalis* [48]. Besides *P*. *gingivalis*, smoking has been suggested to modulate circulating ACPAs by reducing anti-*P*. *gingivalis* titers in non-RA periodontitis patients [49]. Although the number of current smokers in our cohort is low, almost half of the RA patients were former smokers. The results of our cross-sectional study may indicate that the proposed subversion of host immune response by *P*. *gingivalis* is not a critical event. However, the oral microbiome is more than the sum of each bacterium and their interactions are by far not fully understood. Other oral pathogens than *P*. *gingivalis* [8] or the entire subgingival microbiome could have an impact on RA. This has to be investigated in a larger cohort, preferably in a longitudinal setting.

Smoking is known to affect both general and periodontal health and to increase the risk for RA development, particularly in seropositive RA patients [50]. Smokers present more severe periodontal destruction compared to non-smokers [51], which was also true for the current RA population. The fact that subgingival microbiome of smokers in this population differed significantly from that of non-smokers is in line with other reports on periodontitis patients [52], as well as on periodontally healthy individuals [53].

This study involved a very heterogeneous population of RA patients with considerable variety in disease activity, medication, smoking status and oral health. Integrated analysis of microbiological, rheumatological and oral health related factors led to dichotomizing of our study population into two subgingival microbiome community types, CT1 and CT2. RA patients with CT1 included most current smokers, had higher proportion of periopathogenic taxa and lower oral health status compared to patients with CT2, who had healthier microbiome, had higher RA activity and longer disease duration and were more often exposed to prednisolone. This may indicate that CT1 patients may particularly benefit from systematic periodontal therapy approach. Our study suggests that RA therapy and lifestyle factors such as smoking influence subgingival microbiome. Longitudinal, large cohort studies on pre-RA and treatment-naïve RA patients entering therapy are needed to assess the stability of community types and their potential role in RA.

## Conclusions

In RA patients with active disease, anti-inflammatory medication as part of RA therapy was associated with better oral health status and healthier subgingival microbiome compared to that of RA patients in remission, especially those in remission who were current smokers. RA patients in remission with current smoking status may particularly benefit from systematic periodontal treatment program. Current findings suggest a potential role for oral microbial community types in patient stratification for disease outcome prediction and personalized therapy. Longitudinal studies should be conducted to elucidate the relation between RA disease dynamics and subgingival microbiome.

## Supporting information

S1 Supplementary material and methods (DOCX)

S1 Table Categorical variables of selected clinical characteristics of rheumatoid arthritis patients (N = 78). (DOCX)

S2 Table Selected demographic, behavioral and clinical continuous variables of rheumatoid arthritis patients (N = 78). (DOCX)

S3 Table Dataset of all OTUs, subsampled at 5000 reads/sample, including HOMD taxonomy). (XLSX)

S4 Table Significantly discriminatory OTUs between different microbiome sample clusters. (A) Smoking status, (B) Tertiles of PD, (C) Tertiles of PI (D) Tertiles of BoP, (F) RA disease activity, active RA and RA remission, (G) Community type 1 and community type 2, CT1 and CT2.
(XLSX)

S1 Fig Association between dental plaque index (PI) and subgingival microbiome of rheumatoid arthritis (RA) patients. A, nMDS plot based on two-dimensional Bray-Curtis similarity index (stress 0.1991, PERMANOVA: *p* = 0.029, F = 1.7). Pairwise comparisons: the samples with the lowest tertile of the PI (PI tertile 1) vs the samples with the highest PI (PI tertile 3): *p* = 0.023. Samples in the lowest tertile of PI (7–25%) = aqua dots; samples in the middle tertile of PI (25–36%) = purple dots; highest tertile of PI (36–86%) = red dots. B, Boxplots of the most abundant and significant 27 OTUs (S4C Table) that significantly associated with mean PI by linear discriminant analysis effect size (LEfSe) analysis. *P* values are based on Wilcoxon rank-sum test. The boxplots show medians, the error bars indicate 5–95% confidence interval. The connectors show statistically significant differences (*p*<0.05).
(TIF)

S2 Fig Association between bleeding in probing (BoP) and subgingival microbiome of rheumatoid arthritis (RA) patients. A, nMDS plot based on two-dimensional Bray-Curtis similarity index (stress 0.1981, PERMANOVA: *p* = 0.008, F = 1.9). Pairwise comparisons: the samples with the highest tertile of BoP (BoP tertile 3) vs the samples with the lowest BoP (BoP tertile 1): *p* = 0.029 and the samples with the highest tertile of BoP (BoP tertile 3) vs the samples with the moderate BoP (BoP tertile 2): *p* = 0.031). Samples in the lowest tertile of BoP (2–23%) = aqua dots; samples in the middle tertile of BoP (24–35%) = purple dots; highest tertile of PI (35–82%) = red dots. B, Boxplots of the most abundant and significant 21 OTUs (S4D Table) that significantly associated with mean BoP by linear discriminant analysis effect size (LEfSe) analysis. *P* values are based on Wilcoxon rank-sum test. The boxplots show medians, the error bars indicate 5–95% confidence interval. The connectors show statistically significant differences (*p*<0.05).
(TIF)

S3 Fig Relation between the relative abundance of *P*. *gingivalis* in subgingival plaque assessed by 16S rRNA gene sequencing (OTU9 as % of all reads/sample) and by specific *P*. *gingivalis* qPCR probe (% of *P*. *gingivalis* specific PCR over total 16S rDNA/sample). (TIF)
