# Supplementary material for: Subgingival microbiome of rheumatoid arthritis patients in relation to their disease status and periodontal health
Source: PLoS One. 2018 Sep 19;13(9):e0202278. doi: 10.1371/journal.pone.0202278 (PMC6145512; doi:10.1371/journal.pone.0202278)
Supplement: S1 Supplementary material and methods — (DOCX) [file pone.0202278.s001.docx]

# Supplementary materials and methods

## Intra-examiner calibration training

Prior to the study, intra-examiner calibration training in reproducibility of periodontal registration was conducted under supervision of an experienced periodontist (AIB)[1]. Duplicate full-mouth measurements of probing depth (PD), gingival recession (GR) and gingival height (GH) were performed, one hour apart (n = 9). All periodontal measurements were rounded to the nearest millimeter. PD was defined as the distance from the free gingival margin (FGM) to the base of the probable gingival sulcus. The GR was measured as the distance between cemento-enamel junction (CEJ) and FGM if the FGM was located apical to the CEJ. In case that the FGM was located coronal to the CEJ, the GH was measured as the distance from the FGM to the CEJ. Clinical attachment level (CAL) was measured indirectly by calculating PD plus GR or PD minus GH depending on the localization of the FGM. The intra-examiner percentage accuracy and reproducibility for each of the duplicate measurements was calculated separately for each site and analyzed using repeated measures analysis of variance (ANOVA). The intra-class correlation coefficient (ICC) ranged between 0.83 – 0.91 for PD and 0.93 – 0.98 for CAL.

## Oral examination on periodontal status

All RA patients underwent a comprehensive periodontal examination including registration of PD, CAL, bleeding on probing (BoP) and accumulation of dental plaque (PI) assessed at six sites per tooth (disto-buccal, buccal, mesio-buccal, mesio-oral, oral, disto-oral) using a manual periodontal probe with 2 mm increments (PCP-26, Hu-Friedy®, Chicago, IL, USA). Third molars were excluded (except when in position of second molars). The BoP, registered as present or absent, was assessed based on gingival bleeding within 30 sec following periodontal probing to the base of the probable gingival sulcus, as a modification of the method of Ainamo & Bay [2]. Based on the percentage of the dichotomous scores, a gingival BoP index was calculated. Dental plaque was stained with fluorescent disclosing solution (Plaque Test, Ivoclar Vivadent AG, Liechtenstein) and visualized using ultraviolet light (Satelec Mini LED, Kaltenbach & Voigt GmbH, Germany) according to manufacturer’s recommendations. As a supplement to staining, the periodontal probe was used to discriminate between plaque and pellicle. The PI was calculated as percentage of stained tooth surfaces [3]. The degree of tooth mobility was recorded using the Miller index [4]. For multi-rooted teeth the degree of furcation involvement was obtained [5]. After clinical examination, a full mouth series of intra-oral radiographs were taken for patients with PD ≥ 4mm. Minor series containing molar/premolar bitewing radiographs and periapical radiographs of the upper and lower front teeth were taken for patients with PD ≤ 3 mm.

## Sample processing and 16S rDNA amplicon sequencing

The tubes containing subgingival plaque were thawed and centrifuged for 15 min at 15871 x g. The supernatant was removed and the pellet was resuspended in 150 µl sterile Tris-EDTA (TE) buffer and transferred to a 2 ml deepwell plate (Axygen scientific inc, CA, USA). The deepwell plate contained 500 µl 0.1 mm Zyrconia beads, 500 µl phenol (Rotiphenol, Carl Roth GMBH&Co. KG, Germany) and 350 µl lysis buffer (MagMini DNA isolation kit, LCG Genomics Ltd, UK). The deepwell plate was sealed with a silicone lid (Axygen Scientific inc, CA, USA) and placed in a Mini-BeadBeater-96 ( BioSpec products, Bartlesville, OK, USA) for 2 min at 2100 oscillations/min. DNA was extracted using the Mag Mini DNA Isolation Kit. Bacterial DNA concentration after purification was determined by quantitative polymerase chain reaction (qPCR), with primers specific to the bacterial 16S rRNA gene [6].

The V4 hypervariable region of the 16S rRNA gene was amplified as described previously [7] with the adaptation that we performed 33 amplification cycles. Paired-end sequencing (250 cycles) of the DNA was conducted on the MiSeq platform (Illumina, San Diego, CA, USA) at VUmc Cancer Center Amsterdam (the Netherlands). The flow cell was loaded with 7 pmol DNA containing 50 % PhiX (Illumina sequencing control).

## *Porphyromonas gingivalis* specific qPCR

*P. gingivalis* in the samples was quantified in duplicate by monoplex qPCR using the Lightcycler 480-II instrument (Roche Diagnostics, Almere, the Netherlands). The qPCR was carried out in 20 µl reaction mix containing 10 µl Lightcycler® 480 Probes Master (Roche Diagnostics), 4 µl sample, 1.8 pmol forward and reverse primer (F, GCGCTCAACGTTCAGCC, -R, CACGAATTCCGCCTGC) and 0.4 pmol probe (P, Cyan500-CACTGAACTCAAGCCCGGCAGTTTCAA-BBQ) (8). PCR conditions were: 5 min 95°C pre-incubation followed by 45 cycles of detection at 95°C for 10 s, at 60°C for 20 s and at 72°C for 30 s. To quantify the PCR results, a standard curve of *P. gingivalis* strain W83 was generated. Water was used as a negative control for PCR.

## Sequencing data processing

The paired-end sequencing reads were merged, processed and clustered using USEARCH version 8.0.1623 [9]. The sequences were quality filtered using maximum expected error rate of 0.5 (no ambiguous bases allowed) and maximum length of the merged reads of 258. Before clustering, the sorted reads were checked against the Illumina PhiX RTA reference, using both local and global alignment (USEARCH with -id 0.5 -query_cov 0.5) to exclude the possibility that PhiX reads were included during clustering. The merged sequences were clustered into operational taxonomic units (OTUs) using the following adaptations: cluster_otus with ‑uparse_maxdball 1200, only de novo chimera checking, usearch_global with −maxaccepts 8 –maxrejects 64 –maxhits 1. QIIME version 1.8.0 [10] was used to select the most abundant sequence of each OTU as representative sequence and assign a taxonomy to it, using the RDP classifier [11] (min confidence 0.8) and the SILVA 119 database [12]. The alignment of the 97% representative 16S ribosomal DNA sequence set, provided by the QIIME developers, was first trimmed to the V4 region and the alignment was converted to a set of gap-free non-redundant sequences. This set was used to retrain the RDP classifier. To further classify the OTUs, the OTU representative sequences were also assigned a taxonomy using the Human Oral Microbiome Database (HOMD) [13]. The alignment of HOMD 16S rRNA RefSeq version 14.51 was trimmed to the V4 region and used as described above for the SILVA database. The OTUs that originated from quality controls (DNA extraction blanks and PCR blanks) were removed from the OTU table. The OTU table was randomly subsampled to an equal depth per sample using QIIME.

## Statistical analyses

The distribution of the variables was tested using the Shapiro-Wilk test for normality with a critical alpha value of 5%. Mean and standard deviation, and median and range were calculated for continuous variables. The Wilcoxon rank-sum test was applied for not normally distributed continuous variables. Student t-test (for continuous variables) and Pearson chi-square test (for categorical variables) were used to assess differences between the groups. These analyses were performed using the statistical software STATA® version 14.0 for Microsoft® Windows® (StataCorp LP, Texas, USA).

Nonmetric multidimensional scaling (nMDS) plots based on Bray-Curtis distance were used to visualize similarity between groups of samples. Stress value <0.2 (Kruskal’s stress formula 1) was used as an acceptable threshold [14]. One-way permutational multivariate analysis of variance (PERMANOVA) with Bray-Curtis distance measure was used to assess differences in microbiome profiles among different groups of categorical variables and among the groups obtained from continuous variables categorized into tertiles (lowest, middle and highest tertile). If more than two groups were compared, the p-value was corrected using Bonferroni correction. Canonical correspondence analysis (CCA) was used to visualize the relation of microbiome composition with continuous variables. This analysis results in an ordination plot that displays variation in community composition explained by environmental variables and visualized the distribution of samples and individual OTUs in relation to these environmental variables [15]. The significance of the variance of each of the CCA axes was calculated by permuting the data 100 times. To assess microbiome diversity, the Shannon Diversity index was used. All above analyses were performed in PAST, version 3.16 [16].

Linear Discriminant Analysis Effect Size (LEfSe) was used to determine which OTUs contributed to the observed significant differences among different groups of samples in PERMANOVA results [17]. The default settings were used (factorial Kruskal-Wallis test among classes: α = 0.05, threshold on the logarithmic LDA score for discriminative features: 2.0, and the strategy for multi-class analysis was set to be all-against-all). Only OTUs with at least 100 reads were included in the analyses.

Assessment of significant patterns of microbial co-occurrence or mutual exclusion at the genus or higher taxonomic level was performed using CoNet v.1.0b6 (18) and visualized in Cytoscape v. 3.3.0. For this, a dataset of relative abundances of reads at the genus level including the 52 most abundant (average abundance 0.05% or above) genera or higher taxa, and the remaining taxa collectively termed “Others”, was used. An ensemble approach was used, where two measures of correlation (Pearson and Spearman) and two measures of dissimilarity (Bray Curtis and Kullback-Leibler) were combined as described by Faust et al. [18], with a threshold of 0.5 and at least two methods supporting an association. The data matrix was randomized by 100 row-wise permutations and P values were adjusted by Benjamini-Hochberg FDR [19] correction, retaining only *P*<0.05.

## **References**

1. Kingman A, Albandar JM. Methodological aspects of epidemiological studies of periodontal diseases. Periodontol 2000 2002;29:11-30.

2. Ainamo J, Bay I. Problems and proposals for recording gingivitis and plaque. Int Dent J 1975;25(4):229-35.

3. O'Leary TJ, Drake RB, Naylor JE. The plaque control record. J Periodontol 1972;43(1):38.

4. Miller S. Textbook of Periodontia. Philadelphia: Blakiston Company 1938.

5. Hamp SE, Nyman S, Lindhe J. Periodontal treatment of multirooted teeth. Results after 5 years. J Clin Periodontol 1975;2(3):126-35.

6. Ciric L, Pratten J, Wilson M, Spratt D. Development of a novel multi-triplex qPCR method for the assessment of bacterial community structure in oral populations. Environ Microbiol Rep 2010;2(6):770-4.

7. O'Donnell LE, Robertson D, Nile CJ, Cross LJ, Riggio M, Sherriff A, et al. The Oral Microbiome of Denture Wearers Is Influenced by Levels of Natural Dentition. PLoS One 2015;10:e0137717.

8. Boutaga K, van Winkelhoff AJ, Vandenbroucke-Grauls CM, Savelkoul PH. Comparison of real-time PCR and culture for detection of *Porphyromonas gingivalis* in subgingival plaque samples. J Clin Microbiol 2003;41(11):4950-4.

9. Edgar RC. UPARSE: highly accurate OTU sequences from microbial amplicon reads. Nat Methods 2013;10(10):996-8.

10. Caporaso JG, Kuczynski J, Stombaugh J, Bittinger K, Bushman FD, Costello EK, et al. QIIME allows analysis of high-throughput community sequencing data. Nat Methods 2010;7(5):335-6.

11. Cole JR, Wang Q, Cardenas E, Fish J, Chai B, Farris RJ, et al. The Ribosomal Database Project: improved alignments and new tools for rRNA analysis. Nucleic Acids Res 2009;37:D141-5.

12. Pruesse E, Quast C, Knittel K, Fuchs BM, Ludwig W, Peplies J, et al. SILVA: a comprehensive online resource for quality checked and aligned ribosomal RNA sequence data compatible with ARB. Nucleic Acids Res 2007;35(21):7188-96.

13. Chen T, Yu WH, Izard J, Baranova OV, Lakshmanan A, Dewhirst FE. The Human Oral Microbiome Database: a web accessible resource for investigating oral microbe taxonomic and genomic information. Database 2010;2010:baq013.

14. Clarke KR. Non-parametric multivariate analyses of changes in community structure. Aust J Ecol. 1993;18(1):26.

15. Ter Braak CJF. Canonical Correspondence Analysis: A New Eigenvector Technique for Multivariate Direct Gradient Analysis. Ecology 1986;67(5):13.

16. Hammer Ø, Harper DAT, Ryan PD. PAST: paleontological statistics software package for education and data analysis. Palaeontol Electron 2001. Available from: http://palaeo-electronica.org/2001_1/past/past.pdf

17. Segata N, Izard J, Waldron L, Gevers D, Miropolsky L, Garrett WS, et al. Metagenomic biomarker discovery and explanation. Genome Biol 2011;12(6):R60.

18. Faust K, Sathirapongsasuti JF, Izard J, Segata N, Gevers D, Raes J, et al. Microbial co-occurrence relationships in the human microbiome. PLoS Comput Biol 2012;8(7):e1002606.

19. Benjamini Y, Hochberg Y. Controlling the False Discovery Rate - a Practical and Powerful Approach to Multiple Testing. J Roy Stat Soc B Met 1995;57(1):289-300.
