# Supplementary material for: Subgingival microbiome of rheumatoid arthritis patients in relation to their disease status and periodontal health
Source: PLoS One. 2018 Sep 19;13(9):e0202278. doi: 10.1371/journal.pone.0202278 (PMC6145512; doi:10.1371/journal.pone.0202278)
Supplement: S1 Table — (DOCX) [file pone.0202278.s002.docx]

| **S1 Table.** Categorical variables of selected clinical characteristics in rheumatoid arthritis (RA) patients (*N* = 78). | |
| --- | --- |
| Variables | Study population  N (%) |
| Females | 57 (73%) |
| Smoking status |  |
| Never | 29 (37%) |
| Former | 35 (45%) |
| Current | 14 (18%) |
| Periodontitis | 64 (82%) |
| OIDP: presence of complaints | 23 (30%) |
| Active RA disease | 45 (58%) |
| Joint damage | 41 (53%) |
| Seropositivity | 60 (77%) |
| RF^†^ | 41 (55%) |
| ACPAs^‡^ | 55 (71%) |
| Anti- rheumatic medication |  |
| Conventional (C) DMARDs | 33 (42%) |
| Biological (B) DMARDs | 11 (14%) |
| Combination of C & B DMARDs | 30 (38%) |
| Prednisolone | 21 (27%) |
| Values reported as *N* (number) and *%* (percentage) of patients. Periodontitis=mild, moderate and severe periodontitis; OIDP=oral impacts on daily living; Active RA disease=disease activity score (DAS28) ≥2.6; Joint damage=measured by radiographs of the hands and feet and recorded as present or absent; Seropositivity=patients tested positive for rheumatoid factor (RF) and anti-citrullinated protein antibody (ACPAs); DMARDs=disease-modifying anti-rheumatic drugs grouped as follows: conventional DMARDs (methotrexate, leflunomide, hydroxychloroquine, sulfasalazine) and biological DMARDs (tumor necrosis factor (TNF)-inhibitors, B-cell inhibitors, interleukin-6 (IL-6 inhibitors) and a combination of conventional and biological DMARDs.  ^†^ *n* = 74  ^‡^ *n* = 77 | |
