# Supplementary material for: Subgingival microbiome of rheumatoid arthritis patients in relation to their disease status and periodontal health
Source: PLoS One. 2018 Sep 19;13(9):e0202278. doi: 10.1371/journal.pone.0202278 (PMC6145512; doi:10.1371/journal.pone.0202278)
Supplement: S2 Table — (DOCX) [file pone.0202278.s003.docx]

| **S2 Table.** Selected demographic, behavioral and clinical continuous variables in rheumatoid arthritis (RA) patients (N = 78) reported in tertiles. | | | | | | |
| --- | --- | --- | --- | --- | --- | --- |
| Variables | 1^st^ Tertile | | 2^nd^ Tertile | | 3^rd^ Tertile | |
|  | Mean±SD | Median(Range) | Mean±SD | Median(Range) | Mean±SD | Median(Range) |
| Age | 43.6±4.8 | 43.8 (35.1–52.6) | 57.7±3.4 | 57.3 (52.7–62.9) | 69.8±3.9 | 69.6 (63.5–77.6) |
| BMI | 21.7±1.3 | 21.8 (19.4–23.3) | 25.6±1.4 | 25.7 (23.4–27.6) | 31.2±2.7 | 30.5 (28.3–37.3) |
| Teeth | 19.6±6.0 | 23.0 (5–24) | 26.3±0.8 | 27.0 (25–27) | 28.7±1.3 | 28.0 (28–32) |
| PD | 2.4±0.1 | 2.4 (2.2–2.5) | 2.7±0.1 | 2.7 (2.6–2.8) | 3.2±0.4 | 3.0 (2.8–4.4) |
| CAL | 2.5±0.1 | 2.5 (2.4–2.6) | 2.8±0.1 | 2.8 (2.7–3.1) | 3.8±1.0 | 3.5 (3.1–7.0) |
| BoP | 15.9±5.6 | 17.0 (2.0–23.0) | 29.5±3.9 | 29.0 (24.0–35.0) | 48.6±12.9 | 45.0 (36.0–82.0) |
| PI | 18.1±6.1 | 20.0 (7.0–25.0) | 30.2±3.2 | 30.0 (26.0–36.0) | 50.4±14.8 | 47.5 (36.0–86.0) |
| US | 0.12±0.06 | 0.12 (0.03–0.21) | 0.28±0.06 | 0.28 (0.21–0.40) | 0.62±0.22 | 0.53 (0.41–1.20) |
| SS | 1.00±0.23 | 1.05 (0.51–1.44) | 1.73±0.20 | 1.70 (1.45–2.13) | 2.91±0.60 | 2.84 (2.16–4.24) |
| RA onset | 27.1±5.9 | 28.0 (15.0-38.0) | 42.3±3.1 | 43.0 (36.0–47.0) | 59.2±7.2 | 58.0 (48.0-74.0) |
| RA dur | 4.2±2.3 | 4.0 (1.0-8.0) | 12.0±2.4 | 12.0 (9.0–16.0) | 27.8±9.0 | 25.5 (17.0–51.0) |
| DAS28 | 2.0±0.3 | 2.1 (1.1–2.3) | 2.8±0.2 | 2.8 (2.4–3.1) | 4.2±1.0 | 4.1 (3.2–6.7) |
| VAS | 6.7±4.3 | 6.5 (0-14.0) | 24.4±6.9 | 25.0 (15.0–38.0) | 52.9±11.1 | 50.0 (40.0–71.0) |
| MHAQ | 0 | 0 | 0.24±0.1 | 0.25 (0.13–0.38) | 0.80±0.24 | 0.8 (0.5–1.38) |
| ESR | 6.5±2.5 | 7.0 (2.0–10.0) | 15.4±2.8 | 15.0 (11.0–20.0) | 37.2±14.7 | 31.5 (21.0–73.0) |
| CRP | 1.2±0.6 | 1.0 (0.5–2.0) | 4.0±1.2 | 4.0 (3.0–6.0) | 21.6±17.3 | 15.0 (7.0-78.0) |
| RF^†^ | 10.1±0.3 | 10.0 (10-11) | 42.1±25.4 | 40.0 (12-95) | 492.6±547.2 | 241.0 (106–2048) |
| VitD | 54.5±9.0 | 55.5 (32.0-66.0) | 71.5±4.6 | 71.0 (66.0-80.0) | 95.6±11.8 | 92.5 (80.0–119.0) |
| Values reported as mean and SD, standard deviation, and median with range. Age=patient`s age in years at the time point of clinical examination; BMI=body mass index; Teeth=number of teeth; PD=probing depth in mm; CAL=clinical attachment level in mm; BoP=% of sites with bleeding on probing; PI=% of sites with dental plaque; US=unstimulated whole saliva in mg/min; SS=stimulated whole saliva in mg/min; RA onset=patient`s age when diagnosed with RA; RA dur=RA duration in years; DAS28=RA disease activity score; VAS=patient global health assessment score on a visual analogue scale; MHAQ=modified health assessment questionnaire; ESR= erythrocyte sedimentation rate in mm/hr; CRP=C-reactive protein in mg/L; RF=rheumatoid factor; VitD=serum 25-hydroxy vitamin D in nmol/L. | | | | | | |
| ^†^ n = 74 | | | | | | |
