# Supplementary figures and images for: Subgingival microbiome of rheumatoid arthritis patients in relation to their disease status and periodontal health
Source: PLoS One. 2018 Sep 19;13(9):e0202278. doi: 10.1371/journal.pone.0202278 (PMC6145512; doi:10.1371/journal.pone.0202278)

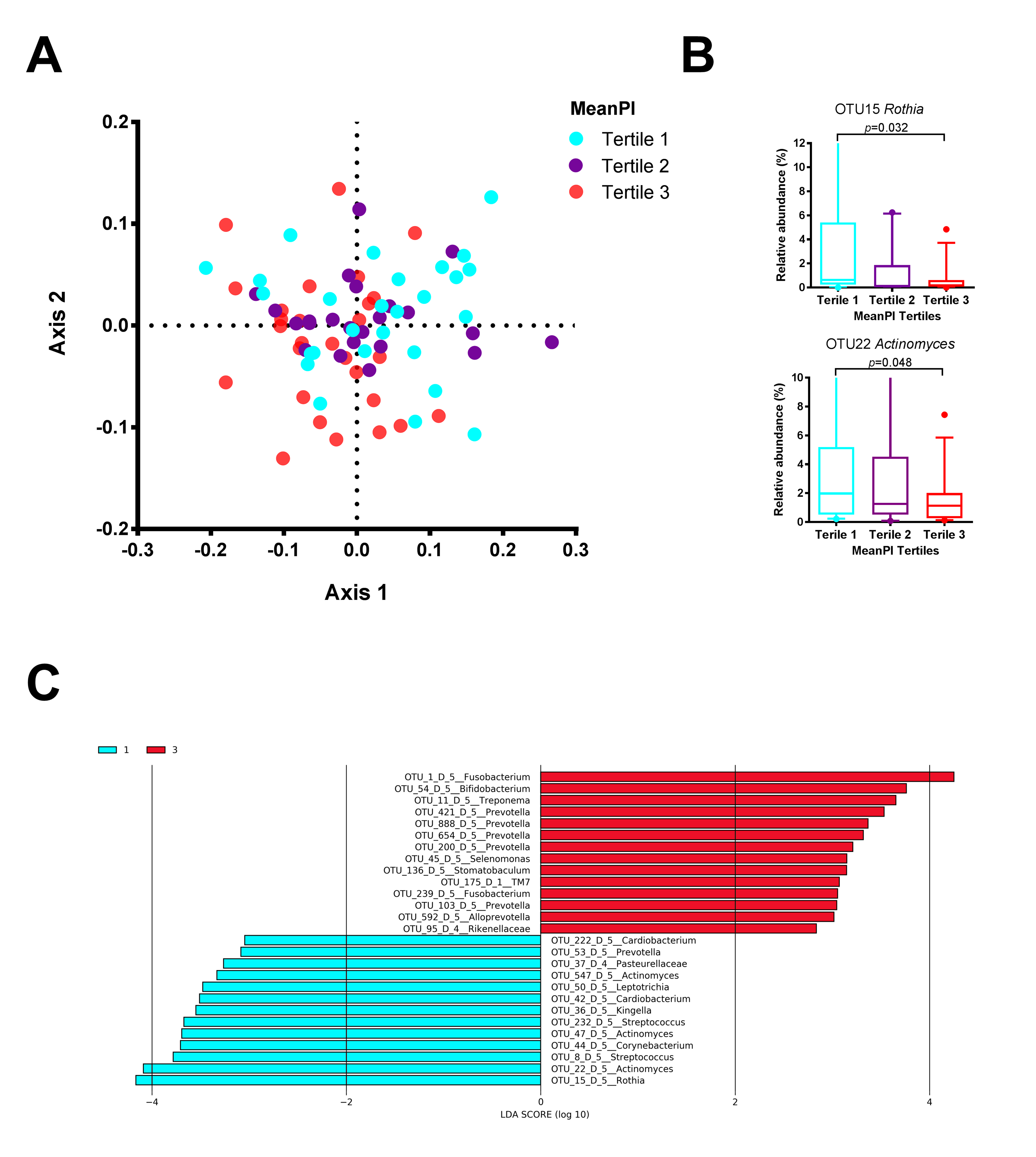

Supplement: S1 Fig — A, nMDS plot based on two-dimensional Bray-Curtis similarity index (stress 0.1991, PERMANOVA: p = 0.029, F = 1.7). Pairwise comparisons: the samples with the lowest tertile of the PI (PI tertile 1) vs the samples with the highest PI (PI tertile 3): p = 0.023. Samples in the lowest tertile of PI (7–25%) = aqua dots; samples in the middle tertile of PI (25–36%) = purple dots; highest tertile of PI (36–86%) = red dots. B, Boxplots of the most abundant and significant 27 OTUs (S4C Table) that significantly associated with mean PI by linear discriminant analysis effect size (LEfSe) analysis. P values are based on Wilcoxon rank-sum test. The boxplots show medians, the error bars indicate 5–95% confidence interval. The connectors show statistically significant differences (p<0.05). (TIF) [file pone.0202278.s006.tif]

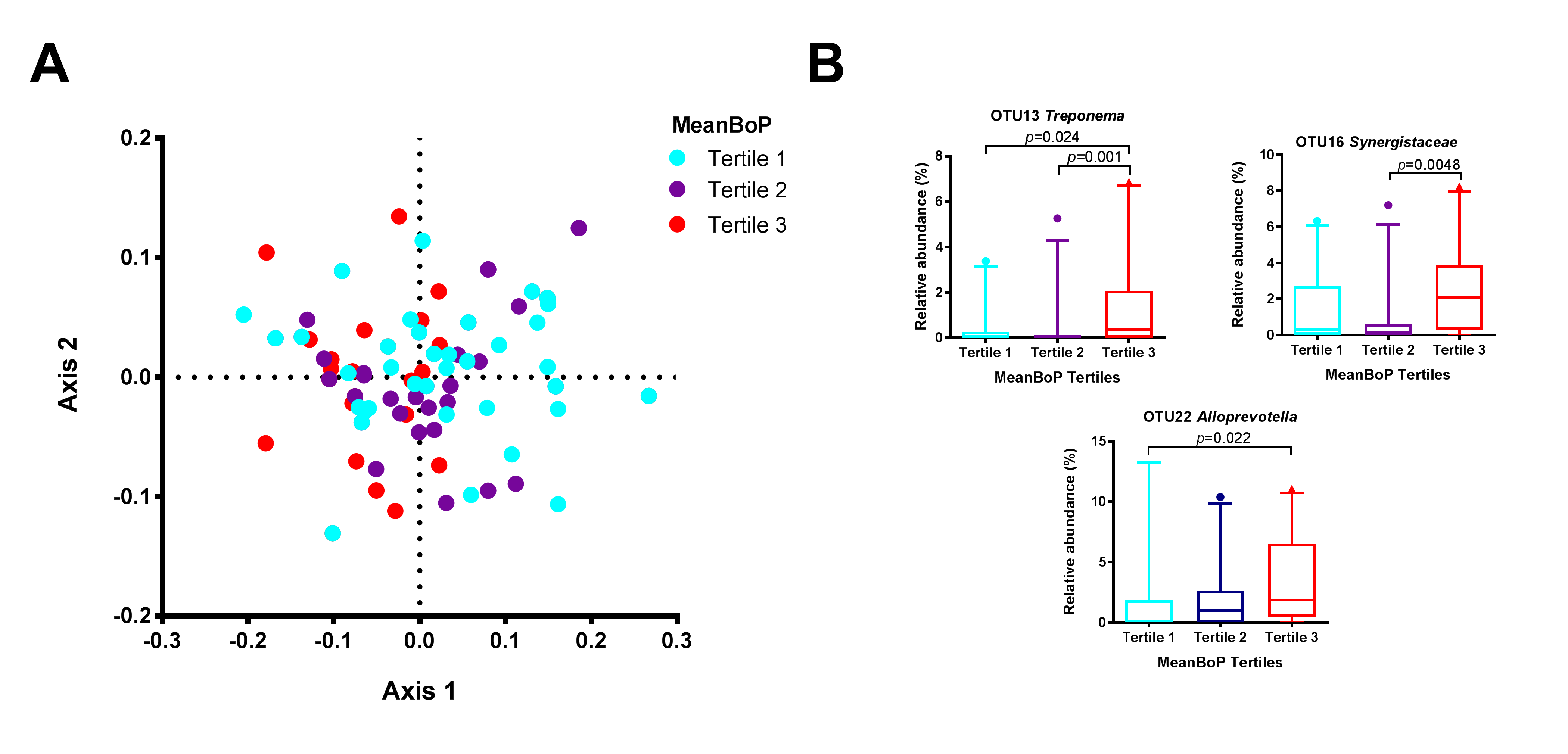

Supplement: S2 Fig — A, nMDS plot based on two-dimensional Bray-Curtis similarity index (stress 0.1981, PERMANOVA: p = 0.008, F = 1.9). Pairwise comparisons: the samples with the highest tertile of BoP (BoP tertile 3) vs the samples with the lowest BoP (BoP tertile 1): p = 0.029 and the samples with the highest tertile of BoP (BoP tertile 3) vs the samples with the moderate BoP (BoP tertile 2): p = 0.031). Samples in the lowest tertile of BoP (2–23%) = aqua dots; samples in the middle tertile of BoP (24–35%) = purple dots; highest tertile of PI (35–82%) = red dots. B, Boxplots of the most abundant and significant 21 OTUs (S4D Table) that significantly associated with mean BoP by linear discriminant analysis effect size (LEfSe) analysis. P values are based on Wilcoxon rank-sum test. The boxplots show medians, the error bars indicate 5–95% confidence interval. The connectors show statistically significant differences (p<0.05). (TIF) [file pone.0202278.s007.tif]

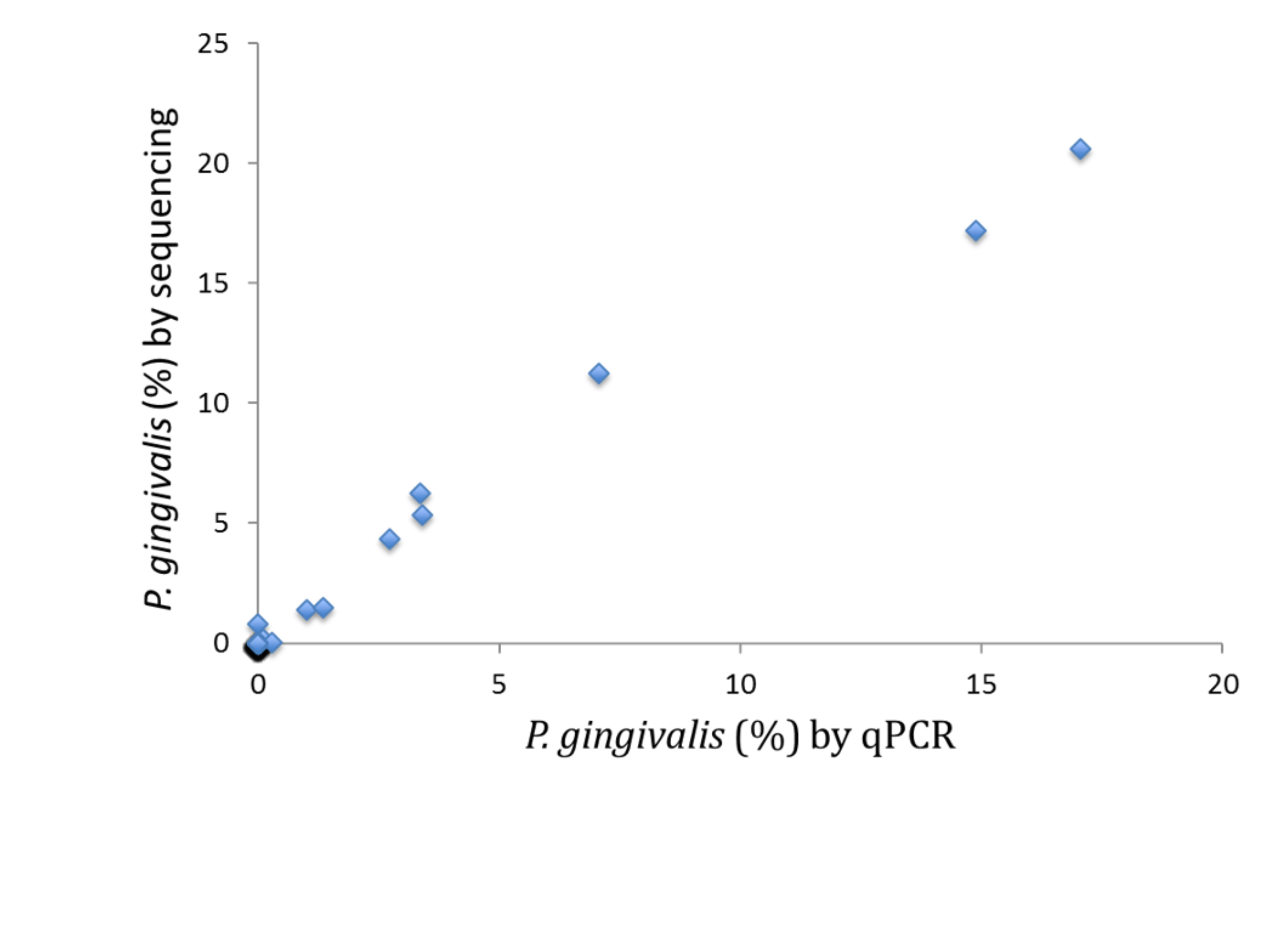

Supplement: S3 Fig — (TIF) [file pone.0202278.s008.tif]
